# Global trends and developments in mindfulness interventions for diabetes: a bibliometric study

**DOI:** 10.1186/s13098-024-01288-x

**Published:** 2024-02-15

**Authors:** Sijia Jiang, Xiaoli Pan, Hansen Li, Yuqin Su

**Affiliations:** 1 College of Physical Education, Chongqing University of Posts and Telecommunications, Chongqing, China; 2 Institute of Sport Science, College of Physical Education, Southwest University, Chongqing, China

**Keywords:** Mindfulness, Intervention, Diabetes, Bibliometrics, Web of Science Core Collection

## Abstract

**Background:**

Diabetes is a metabolic disorder posing a global threat to health. Many scholars are dedicated to developing non-pharmacological therapies, and mindfulness intervention is among the potentially effective approaches. Due to the rapid increase in relevant research in recent years, along with the diverse focus and interventions used in studies, it has become challenging for practitioners to quickly comprehend the key features of this field and the directions worth paying attention to. Bibliometric analysis, in response, can help scholars understand this field and identify points of interest.

**Methods:**

Publications related to mindfulness intervention in diabetes from the establishment of the Web of Science Core Collection (WOSCC) to September 2023 were searched. We employed four bibliometric techniques: General Analysis of Publications, Collaborative Network Analysis, Co-citation Analysis, and Keyword Analysis. The CiteSpace 6.1.R was used to analyze the literature with the strongest citation bursts, while VOSviewer 1.6.13 was used to provide visualizations of publicly available data by analyzing co-citations or co-authorship affiliations.

**Results:**

We found a total of 387 articles. The results indicate that research on this topic has been steadily increasing over time. The United States is the top producer of relevant publications, with Tilburg University being the institution that publishes the most articles. The journal “Mindfulness” has the highest publication count. In the collaborative network analysis, the United States emerged as the main hub for global cooperation in this research field, contributing 182 articles with a total of 5872 citations. The journal “Diabetes Care” was frequently cited and played a central role. The keyword analysis revealed that researchers have shown a strong interest in how mindfulness interventions affect the mental health of diabetic individuals. Additionally, there is a focus on studying elderly diabetic groups and exploring how mindfulness interventions impact metabolic diseases. These areas are currently the main research priorities.

**Conclusion:**

Our findings demonstrate the current trend and hotspots in mindfulness intervention and offer some directions for future research.

## Introduction

Diabetes is a global health problem that leads to mortality and morbidity. As of September 2023, 527 million people worldwide have diabetes, and this number is predicted to exceed 780 million by year 2045 [1]. Diabetes is a chronic hyperglycemic disease, traditionally classified as type 1 diabetes (T1DM) and Type 2 diabetes (T2DM) [2]. Type 1 diabetes usually results in absolute insulin deficiency due to autoimmune beta cell destruction, and patients need daily insulin injections to control blood sugar levels [3]. Type 2 diabetes is caused by the gradual loss of beta cell insulin secretion in the case of insulin resistance and is the most common type of diabetes, accounting for more than 90% of all diabetics [4]. At present, the academic community has not found an effective medical treatment for T1DM, but the occurrence of T2DM can be delayed or prevented through intervention on its causes and symptoms.

There is a strong relationship between diabetes and mental health, with studies showing that anxiety disorders and depression often co-exist with diabetes [5]. People with diabetes often have higher levels of chronic psychological stress, which can lead to increased cortisol levels, elevated blood sugar levels, insulin disorders [6–8]. These adverse effects may in turn exacerbate the psychological problems of patients. Since diabetes is a complex and chronic disease, many countermeasures along with standard medical treatment, such as proper diet, physical activity, self-management, and psychological intervention may also improve the outcome of diabetes [9, 10].

Mindfulness is a activity that emphasizes the focus on the experience of the present moment and being non-judgmental, open, and accepting of whatever comes up internally or externally [11]. Mindfulness can be divided into two general types, namely Mindfulness-based stress reduction (MBSR) and Mindfulness-based Cognitive therapy (MBCT). Mindfulness has been proven to be one of the effective interventions for diabetes. Several reviews have summarized the effectiveness of mindfulness intervention in diabetes, suggesting that mindfulness is effective in improving the mental conditions of diabetes patients [12–15]. Meanwhile, mindfulness may reduce the HbA2c levels in patients with T1DM or T1DM and thereby improve their blood glucose control. Bibliometric analysis is a method grounded in data retrieval and statistical approaches, employed to quantitatively review a large number of articles within a specific research area, thereby uncovering its hotspots and trends [16]. CiteSpace and VOSviewer represent crucial tools in the composition of literature information visualization software [17]. This approach, unlike traditional literature reviews and systematic reviews, provides multiple perspectives in a more intuitive manner [18]. The methodology delineates the hotspots of a research field and predicts emerging research topics by summarizing the volume of citations and publications, as well as the prominence of keywords and thematic terms. In recent years, such an analysis has been widely used in various psychosomatic intervention topics, such as Tai Chi [19], Qigong [20], and yoga [21].

Since increasing studies have focused on the health benefits of mindfulness intervention in diabetes and a bibliometrics analysis is still lacking [19, 22], this paper employs bibliometric analysis to thoroughly understand the research on mindfulness interventions for diabetes. This approach will enable practitioners, therapists, and researchers to better comprehend the field, and to more fully grasp the current research trends, the impact of studies, and scientific collaborations. The objective is to provide quantitative evidence that will guide future research endeavors.

## Method

### Data sources and retrieval strategies

The data for the study were sourced from the Web of Science Core Collection (WOSCC). WOSCC is produced by Thomson and contains a vast amount of literature information, commonly used for bibliometric analysis. It offers comprehensive and multidisciplinary information for statistical analysis, particularly in the fields of diabetes and public health [23–25]. To ensure the selection of high-quality academic journals, we only considered the WOSCC, a digital bibliometric platform recognized internationally by researchers as having high-quality standards [26]. It includes over 21,100 peer-reviewed scholarly journals, published globally across more than 250 disciplines in sciences, social sciences, and arts & humanities. The availability of citation data makes WOSCC data suitable for bibliometric analysis, including co-citation analysis [27]. Our search formula was as follows: {[ALL = ("diabetea") OR ALL = ("diabetic") OR ALL = ("antidiabetic")] AND [ALL = ("Mindfulness") OR ALL = ("mindful")}. We limited document types to articles and language to English.

### Data collection

We downloaded the literature information and kept a complete record of citations and references. With the aid of CiteSpace 6.1.R, we analyzed the literature with the strongest citation bursts, while VOSviewer 1.6.13 was used to provide visualizations of publicly available data by analyzing co-citations or co-authorship affiliations. We employed four bibliometric techniques: General Analysis of Publications, Collaborative Network Analysis, Co-citation Analysis, and Keyword Analysis. We collected the following bibliometric indicators: annual research output, total citations, citation frequency, publication countries, journals and institutions, as well as data on the top ten most cited articles.

### Data analysis

We used the VOSviewer to display a collaborative network of countries and high-frequency keywords, with node types set to countries and all keywords in turn, and different threshold keywords for only the top 10 countries and the top 60 countries. We selected the node weight strength of the country node and the node weight of the keyword on the document based on the total link. We visualized the time line of keyword use CiteSpace and burst keywords, set 2005–2023 since the publication of the first relevant literature, set the time slice to 2 years, and the node type has been set to keyword. We used a network clipping to simplify secondary wiring while the rest of the parameters were set to default settings.

## Result

### General analysis of publications

#### The growth trend of publications

**Fig. 1:**
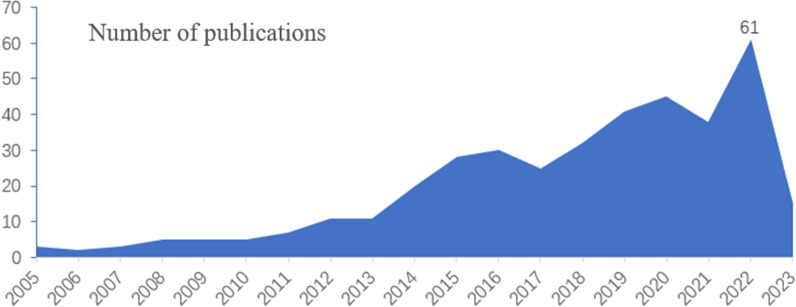
Statistics on the number of articles published on mindfulness-based interventions in diabetes, 2005–2023

Analysis of publication year and chronological distribution can indicate the development of a specific research field [28]. We searched and looked through the WOS core database, finding 411 pieces of literature. After careful screening, we excluded 24 pieces that were not relevant, leaving us with 387 pieces of literature. Figure 1 shows the trend of the number of published documents related to mindfulness intervention in diabetes in the core database of Web of Science over time. Between 2005 and 2010, there were very few studies, with less than 10 published each year. However, from 2011 to 2021, the annual output showed a fluctuating upward trend. In 2022, there was a peak with 61 publications, and as of September 2023, a total of 387 papers related to using mindfulness for diabetes intervention have been published. Although the yearly publication rate stayed below 100, this trend indicates that research on diabetes mindfulness interventions will continue to grow steadily in the future, receiving increasing academic attention.

#### Analysis of citations of publications

Table 1 displays the top 15 articles that have received the most citations in the context of using mindfulness for diabetes intervention. Among these, the article by Gregg [29] and colleagues (published in the Journal of Consulting and Clinical Psychology in 2007) titled “Improving Diabetes Self-Management through Acceptance, Mindfulness, and Values” stands out as the most frequently referenced. It has been cited 430 times. This research involved a randomized controlled trial (RCT) with type 2 diabetes patients from a low-income community. This study showed that a therapy approach focused on mindset positively impacted daily self-management and the HbA(1C) index. Patients in an Acceptance and Commitment Therapy (ACT) state are particularly more likely to utilize mindfulness skills, achieving better diabetes self-care.

**Table 1:** The 15 most cited articles

Rank	Authors	Citations	Journal	Title
1	Gregg et al. (2007)	430	Journal of Consulting and Clinical Psychology	Improving diabetes self-management through acceptance, mindfulness, and values: a randomized controlled trial
2	Abbott et al. (2014)	169	Journal of Psychosomatic Research	Effectiveness of mindfulness-based stress reduction and mindfulness based cognitive therapy in vascular disease: a systematic review and meta-analysis of randomised controlled trials
3	Kubzansky et al. (2018)	167	Journal of The American College of Cardiology	Positive psychological well-being and cardiovascular disease
4	Rosenzweig et al. (2007)	127	Alternative Therapies in Health and Medicine	Mindfulness-based stress reduction is associated with improved glycemic control in type 2 diabetes mellitus: a pilot study
5	van Son et al. (2013)	99	Diabetes Care	The effects of a mindfulness-based intervention on emotional distress, quality of life, and HbA(1c) in outpatients with diabetes (DiaMind)
6	Tovote et al. (2014)	97	Diabetes Care	Individual mindfulness-based cognitive therapy and cognitive behavior therapy for treating depressive symptoms in patients with diabetes: results of a randomized controlled trial
7	Hartmann et al. (2012)	95	Diabetes Care	Sustained effects of a mindfulness-based stress-reduction intervention in type 2 diabetic patients design and first results of a randomized controlled trial (the Heidelberger diabetes and stress-study)
8	Creswell et al. (2019)	92	Psychosomatic Medicine	Mindfulness training and physical health: mechanisms and outcomes
9	Miller et al. (2014)	87	Health Education & Behavior	Comparison of a mindful eating intervention to a diabetes self-management intervention among adults with type 2 diabetes: a randomized controlled trial
10	Merkes et al. (2010)	77	Australian Journal of Primary Health	Mindfulness-based stress reduction for people with chronic diseases
11	Schmidt et al. (2018)	72	Diabetic Medicine	Systematic review and meta-analysis of psychological interventions in people with diabetes and elevated diabetes-distress
12	Zhang et al. (2021)	70	British Medical Bulletin	Mindfulness-based interventions: an overall review
13	Shrestha et al. (2015)	70	Cochrane Database of Systematic Reviews	Workplace interventions for reducing sitting at work
14	Castelnuovo et al. (2016)	63	Frontiers in Psychology	Psychological treatments and psychotherapies in the neurorehabilitation of pain: evidences and recommendations from the Italian consensus conference on pain in neurorehabilitation
15	van Son et al. (2014)	53	Journal of Psychosomatic Research	Mindfulness-based cognitive therapy for people with diabetes and emotional problems: long-term follow-up findings from the DiaMind randomized controlled trial

The work by Abbott et al. [30] (published in 2014) titled “Effectiveness of Mindfulness-Based Stress Reduction and Mindfulness-Based Cognitive Therapy in China: A Systematic Review and Meta-Analysis of Randomized Controlled Trials” and Kubzansky et al. [31] (published in 2018) titled “Positive Psychological Well-being and Cardiovascular Disease” rank second and third with 169 and 167 citations, respectively.

The journal that appears most frequently as a source for these highly-cited articles is the Journal of Consulting and Clinical Psychology (JCCP). JCCP publishes original papers covering various aspects of clinical and clinical psychology, encompassing studies of diverse clinical populations, therapeutic approaches, and psychosocial factors related to health behaviors.

#### Countries with the highest publication productivity

The total number of publications, total citations, and CP (citations/publications) can reflect the impact of published papers in leading countries [32]. In the realm of using mindfulness to intervene in diabetes, the top 10 countries with the most published papers include the United States, the United Kingdom, Australia, the Netherlands, China, Canada, Germany, Denmark, New Zealand, and France (as shown in Table 2). Notably, the United States leads the pack by contributing 48 percent of the publications, significantly more than the other nine countries. The United States, with a substantial 5872 total citations, outperforms other countries by a significant margin. The United Kingdom follows with 862 citations, while the Netherlands holds the third position with 953 citations. In terms of CP, France takes the lead with an score of 36.11, trailed by the United States (32.26), the Netherlands (31.77), and Australia (26.53).

**Table 2:** Top 10 countries by publication productivity

Label	Publications	Citations	Citations/publication	Leading institute of each country (publications)
USA	182	5872	32.26	North Carolina State University (12)
England	41	862	21.02	King’s College London (7)
Australia	34	902	26.53	Deakin University (12)
Netherlands	30	953	31.77	Tilburg University (15)
China	26	270	10.38	The Chinese University of HongKong (4)
Canada	22	324	14.73	University of British Columbia (4)
Germany	13	266	20.46	Ruprecht Karls University Heidelberg (5)
Denmark	10	145	14.50	University of Southern Denmark (7)
New Zealand	10	250	25.00	University of Auckland (8)
France	9	325	36.11	Institut National de la Sante et de la Recherche Medicale (6)

#### The organization with the highest publication productivity

Five out of the top ten most active institutions in the area of using mindfulness for diabetes interventions are situated in the United States, as demonstrated in Table 3. Among these, the leading three institutions are Tilburg University, Deakin University, and the University of North Carolina. Regarding total citations, Tilburg University holds the top spot with 564 citations, followed by the University of California, San Francisco with 551 citations, and Ohio State University with 546 citations. In terms of citations per publication (CP), Ohio State University leads the pack with a substantial score of 78.00, followed by the University of California, San Francisco with 61.22, and the University of Amsterdam with 43.00.

**Table 3:** Top 10 organizations in publication productivity

Label	Publications	Citations	Citations/publication	URL
Tilburg University	15	564	37.60	www.tilburguniversity.edu
Deakin University	12	513	42.75	www.deakin.edu.au
University of North Carolina	12	515	42.92	www.unc.edu
Brown University	9	311	34.56	www.brown.edu
University of California, San Francisco	9	551	61.22	www.ucsf.edu
University of Pittsburgh	9	132	14.67	www.pitt.edu
University of Auckland	8	236	29.50	www.auckland.ac.nz
King’s College London	7	68	9.71	www.kcl.ac.uk
Ohio State University	7	546	78.00	www.osu.edu
University of Amsterdam	7	301	43.00	www.uva.nl

#### The journal with the highest publication productivity

Table 4 shows the top 20 journals regarding the total number of articles related to mindfulness interventions for diabetes, citations, and the number of citations attributed to articles from each journal. The journal “Mindfulness,” which specializes in mindfulness studies, leads the pack with the highest number of published articles (15). It is followed by “Diabetes” (12) and “Diabetic Medicine” (10). When it comes to total citations, “Diabetes Care” takes the top spot with 416 citations, followed by “Mindfulness” (317) and the “Journal of Psychosomatic Research” (312). As for CA, the “Journal of Diabetes Care” ranks first with an impressive score of 104.00, trailed by the “Journal of Psychosomatic Research” (62.40) and the “Journal of Behavioral Medicine” (36.20). Notably, among the top 20 journals, only five articles have received more than 100 citations. From the journal analysis, we observed that researchers published their articles in quality journals with good impact factors. Notably, most of the journals where these articles were published are related to diabetes, mindfulness, and public health, except for “PLOS One,” which is a multidisciplinary journal.

**Table 4:** The top 20 journals by publication productivity

Label	Article number	Citations	Citations/article	Most cited article	Citations of most cited article	Journals IF and ranking	Journals ISSN	Journals URLs
Mindfulness	15	317	21.13	Esther et al. (2014)	149	3.801 (Q2)	1868-8527	https://www.springer.com/12671
Diabetes	12	1	0.08	Nagel et al. (2018)	1	12.30 (Q1)	0012-1797	http://diabetes.diabetesjournals.org/
Diabetic Medicine	10	191	19.10	Schmidt et al. (2018)	72	6.90 (Q3)	0742-3071	http://onlinelibrary.wiley.com/journal/10.1111/(ISSN)1464-5491
Appetite	8	240	30.00	Forman and Butryn (2015)	126	8.10 (Q1)	0195-6663	http://www.journals.elsevier.com/appetite/
Pediatric Diabetes	6	36	6.00	Ellis et al. (2019)	19	5.90 (Q1)	1399-543X	http://onlinelibrary.wiley.com/journal/10.1111/(ISSN)1399-5448
Plos One	6	56	9.33	Shayeghian et al. (2016)	35	6.00 (Q1)	1932-6203	http://www.plosone.org/home.action
Journal of Contextual Behavioral Science	6	30	5.00	Roche et al. (2019)	19	6.90 (Q1)	2212-1447	https://www.journals.elsevier.com/journal-of-contextual-behavioral-science
International Journal of Behavioral Medicine	6	31	5.17	Chan et al. (2016)	16	4.20 (Q3)	1070-5503	https://www.springer.com/12529
Journal of Psychosomatic Research	5	312	62.40	Abbott et al. (2014)	169	6.40 (Q2)	0022-3999	http://www.journals.elsevier.com/journal-of-psychosomatic-research/
Complementary Therapies in Medicine	5	102	20.40	Shomaker et al. (2017)	46	7.20 (Q3)	0965-2299	http://www.journals.elsevier.com/complementary-therapies-in-medicine/
Journal of Behavioral Medicine	5	181	36.20	Garland et al. (2012)	131	5.40 (Q3)	0160-7715	https://www.springer.com/10865
Primary Care Diabetes	5	74	14.80	Pedersen et al. (2017)	33	3.90 (Q3)	1751-9918	http://www.primary-care-diabetes.com/
Annals of Behavioral Medicine	5	2	0.40	Woods-Giscombe et al. (2016)	2	6.50 (Q2)	0883-6612	https://academic.oup.com/abm
Diabetes Care	4	416	104.00	Friis et al. (2016)	125	27.80 (Q1)	0149-5992	http://care.diabetesjournals.org/
Current Diabetes Reports	4	27	6.75	DiNardo et al. (2012)	13	10.70 (Q2)	1534-4827	https://www.springer.com/11892
Diabetes Research and Clinical Practice	4	116	29.00	Massey et al. (2019)	41	10.50 (Q3)	0168-8227	http://www.journals.elsevier.com/diabetes-research-and-clinical-practice/
BMC Public Health	4	56	14.00	van Son et al. (2011)	27	6.10 (Q2)	1471-2458	http://bmcpublichealth.biomedcentral.com
Contemporary Clinical Trials	4	79	19.75	Gross et al. (2017)	32	3.20 (Q3)	1551-7144	http://www.journals.elsevier.com/contemporary-clinical-trials/
Evidence-based Complementary and Alternative Medicine	4	78	19.50	Naliboff et al. (2008)	39	3.50 (Q4)	1741-427X	https://www.hindawi.com/journals/ecam
BMJ Open Diabetes Research & Care	3	11	3.67	Guo et al. (2019)	9	6.90 (Q2)	2052-4897	https://drc.bmj.com/

### Collaborative network analysis

In the visualized results, each node represents a project, which can be an institution or organization. The size of these nodes shows how many papers that project has published. The lines connecting the nodes represent collaborations between projects, and the thickness of these lines shows how strong their collaboration is [33].

#### Analysis of inter-country/regional collaboration networks

**Fig. 2:**
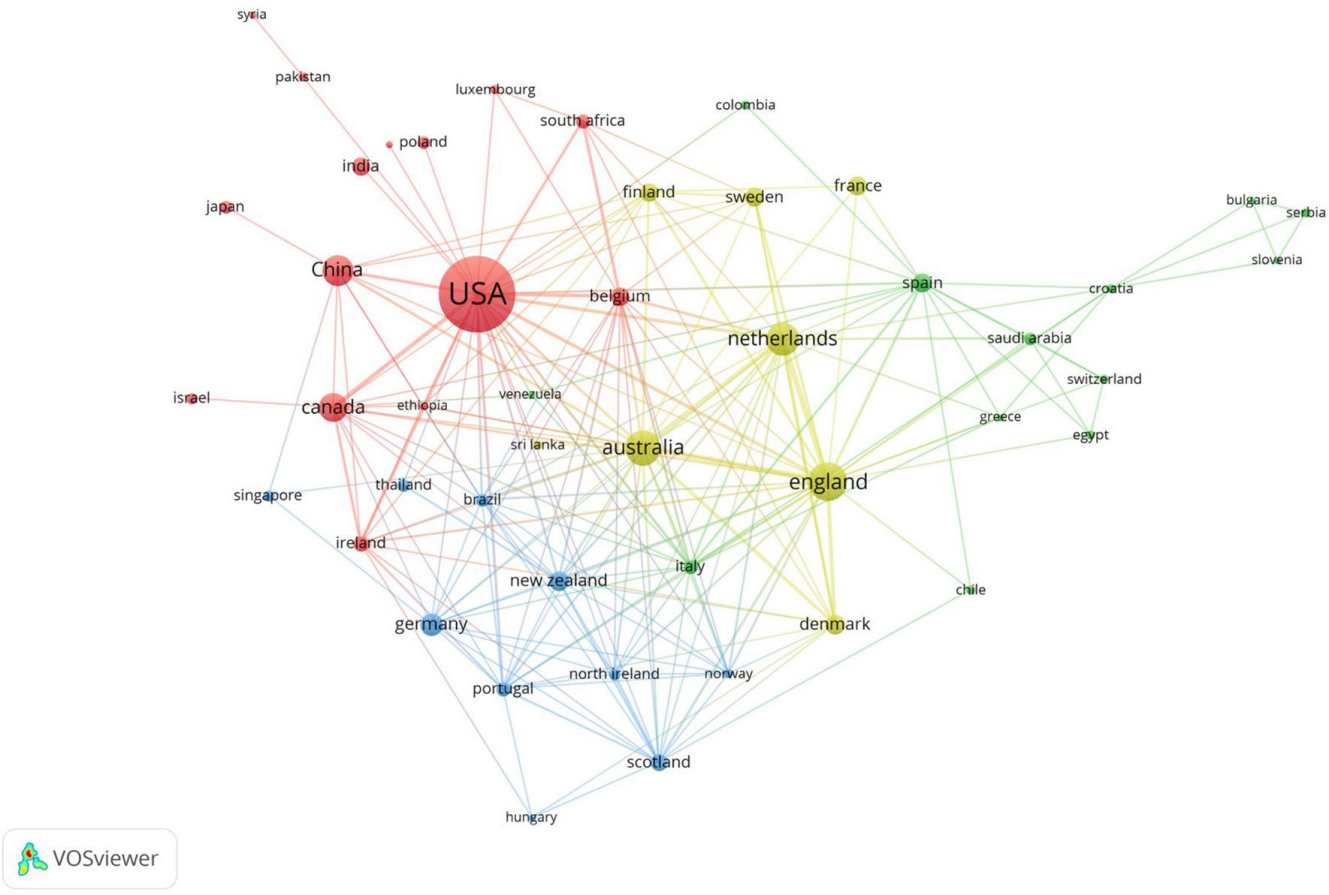
Analysis of inter-country/regional collaboration networks

The strength of connections, the number of references, and the number of citations can indicate the extent of collaboration between countries [33]. Figure 2 displays the collaboration network among countries and regions using mindfulness for diabetes intervention. We analyzed 55 countries with a publication frequency greater than 1, setting a connection strength threshold of 0.

Regarding connection strength, the United States is the central hub for global cooperation in this field, with 182 articles and a substantial 5872 citations. Its international collaboration surpasses that of other countries. Following the United States are the United Kingdom (with 41 references and 862 citations), Australia (with 34 references and 902 citations), the Netherlands (with 30 references and 953 citations), Canada (with 22 references and 324 citations), New Zealand (with ten references and 250 citations), Italy (with five references and 181 citations), Denmark (with ten references and 14 citations), Belgium (with eight references and 97 citations), and Scotland (with seven references and 44 citations).

#### Inter-agency collaboration network analysis

**Fig. 3:**
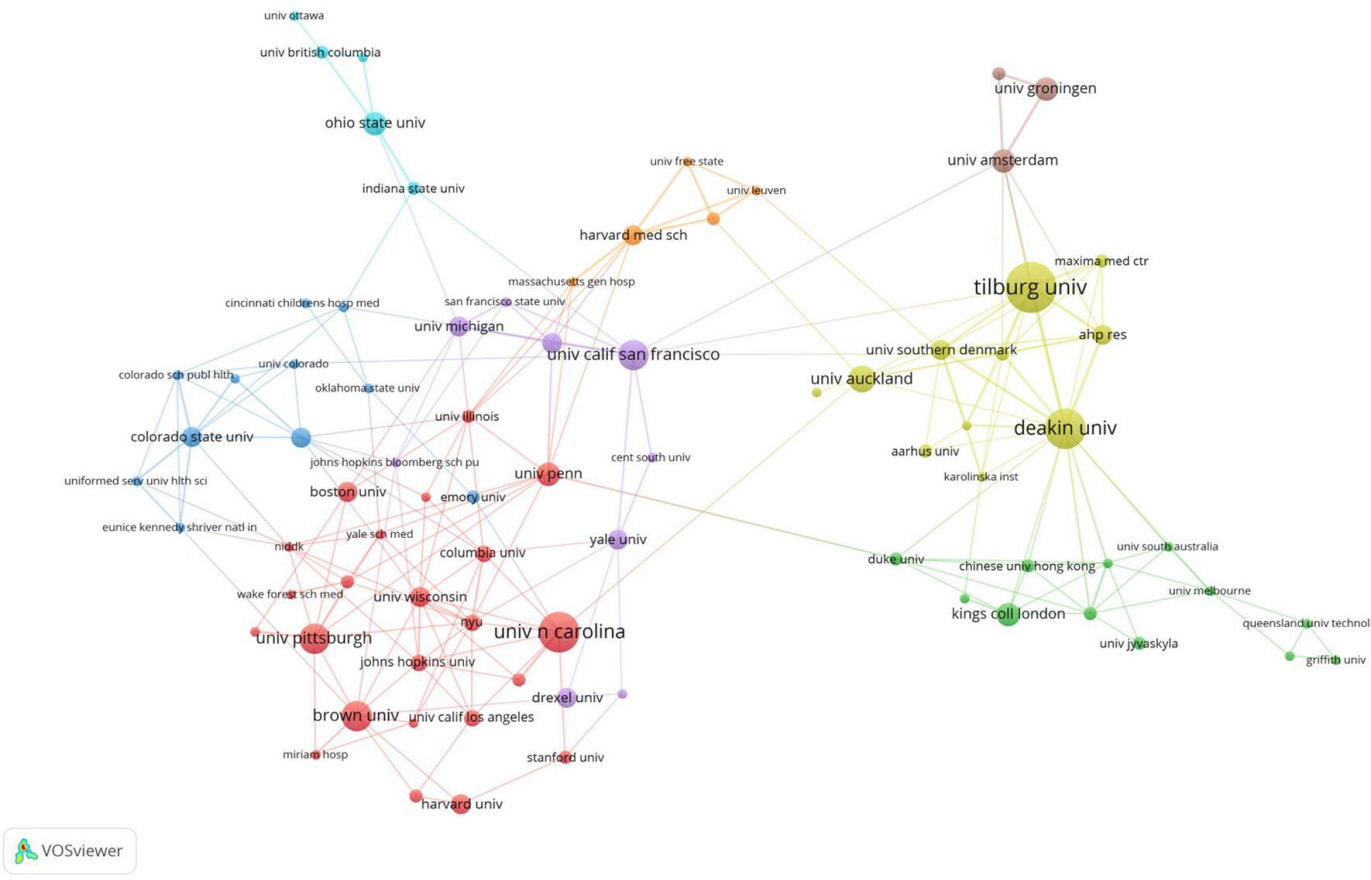
Inter-agency collaboration network analysis

The study applied a threshold of 3, identifying 87 institutions out of the initial 749 that met the threshold criteria. Figure 3 illustrates the collaborative network among these institutions (with a connection strength threshold set at 1). Regarding connection strength, Tilburg University takes the central role in global collaboration, having a strength of 30 and garnering 564 citations. Following closely is Deakin University, with a strength of 38 and 513 citations. Other notable institutions in the collaboration network include the University of Southern Denmark (with a strength of 21 and 75 citations), AHP Research (with a strength of 19 and 123 citations), Radboud University Nijmegen (with a strength of 18 and 176 citations), University of California, San Francisco (with a strength of 16 and 551 citations), University of California, San Francisco (with a strength of 15 and 110 citations), Harvard Medical School (with a strength of 15 and 236 citations), University of Amsterdam (with a strength of 15 and 301 citations), and Northwestern University (with a strength of 13 and 417 citations).

### Co-citation analysis

When two items, like articles, are referenced together in a citation, they are cited simultaneously. This simultaneous citation count can be employed to gauge the similarity and relevance between articles [34]. We can examine journal co-citation, article co-citation, and keyword co-citation based on the specific content of various projects.

#### Journal co-citation analysis

**Fig. 4:**
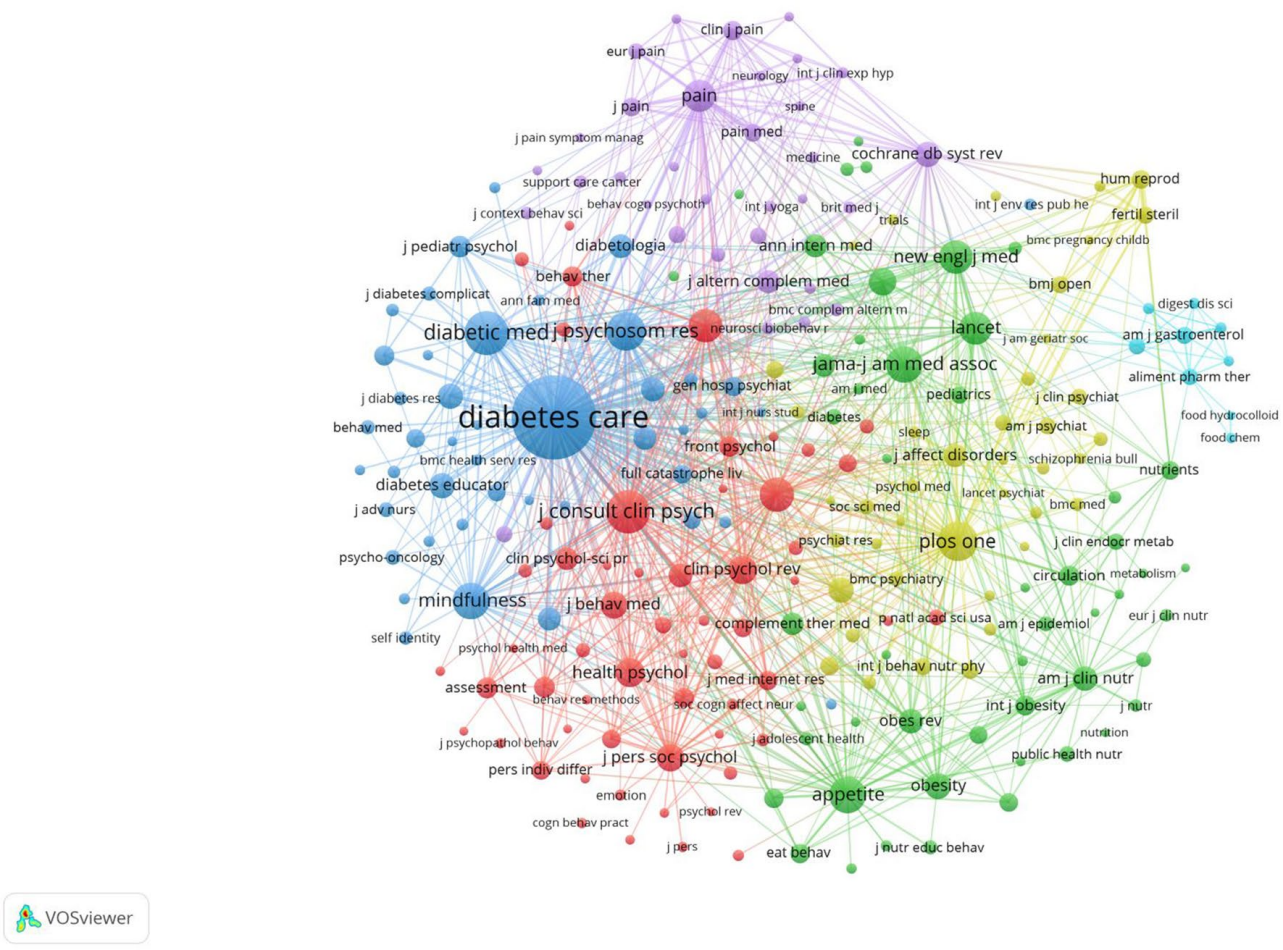
Network diagram of journal co-citation analysis from 2005 to 2023

By analyzing co-cited literature, we can build a knowledge foundation for that particular field. The research frontier is essentially a compilation of citations within these repositories [35]. Table 5 presents the outcomes of our analysis on journal co-citation, while Fig. 4 offers a visual representation of the network of co-citations among journals. In this network, VOSviewer identified 4212 journals, and Fig. 4 focuses on 119 of them. Notably, “Diabetes Care” is the most frequently cited journal and holds a central position in this network, followed by “Diabetic Medicine” and the “Journal of Consulting and Clinical Psychology.”

**Table 5:** The top 20 journals in terms of co-citations

Rank	Journal	Citations	Total link strength	Journals IF and Q	Journals ISSN	Journals URLs
1	Diabetes Care	816	24,339	27.80 (Q1)	0149-5992	http://care.diabetesjournals.org/
2	Diabetic Medicine	250	8154	6.90 (Q1)	0742-3071	http://onlinelibrary.wiley.com/journal/10.1111/(ISSN)1464-5491
3	Journal Consulting and Clinical Psychology	243	10,132	9.10 (Q1)	0022-006X	https://www.apa.org/pubs/journals/ccp/
4	Plos One	207	10,523	6.00 (Q1)	1932-6203	http://www.plosone.org/home.action
5	Journal of Psychosom Research	199	8246	6.40 (Q2)	0022-3999	http://www.journals.elsevier.com/journal-of-psychosomatic-research/
6	Jama- Journal of the American Medical Association	183	7169	45.40 (Q1)	0098-7484	http://jama.ama-assn.org/
7	Appetite	177	7286	8.10 (Q1)	0195-6663	http://www.journals.elsevier.com/appetite/
8	Mindfulness	177	7046	3.801 (Q2)	1868-8527	https://www.springer.com/12671
9	Psychosom Medicine	160	7633	5.50 (Q2)	0033-3174	http://journals.lww.com/psychosomaticmedicine/pages/default.aspx
10	Behavour Research and Therapy	155	6765	7.90 (Q1)	0005-7967	https://www.journals.elsevier.com/behaviour-research-and-therapy
11	New England Journal of Medicine	154	5863	134.40 (Q1)	0028-4793	http://www.nejm.org/
12	Lancet	147	6683	133.20 (Q1)	0140-6736	http://www.thelancet.com/
13	Pain	142	9612	12.50 (Q1)	0304-3959	https://journals.lww.com/pain/pages/default.aspx
14	Health Psychology	121	5257	6.10(Q1)	0278-6133	http://www.health-psychology.com/
15	Clinical Psychology Review	115	6018	18.90 (Q1)	0272-7358	https://www.journals.elsevier.com/clinical-psychology-review
16	Bmj-british Medical Journal	107	4854	15.30 (Q1)	1756-1833	http://www.bmj.com/
17	Journal of Personality and Social Psychology	107	5693	11.70 (Q1)	0022-3514	https://www.apa.org/pubs/journals/psp/
18	Journal of Behavioral Medicine	103	3885	5.40 (Q2)	0160-7715	https://www.springer.com/10865
19	Obesity	98	4442	11.80 (Q1)	1930-7381	http://onlinelibrary.wiley.com/journal/10.1002/(ISSN)1930-739X
20	Diabetes Research and Clinical Practice	95	2863	10.50 (Q2)	0168-8227	http://www.journals.elsevier.com/diabetes-research-and-clinical-practice/

#### Reference co-citation analysis

**Fig. 5:**
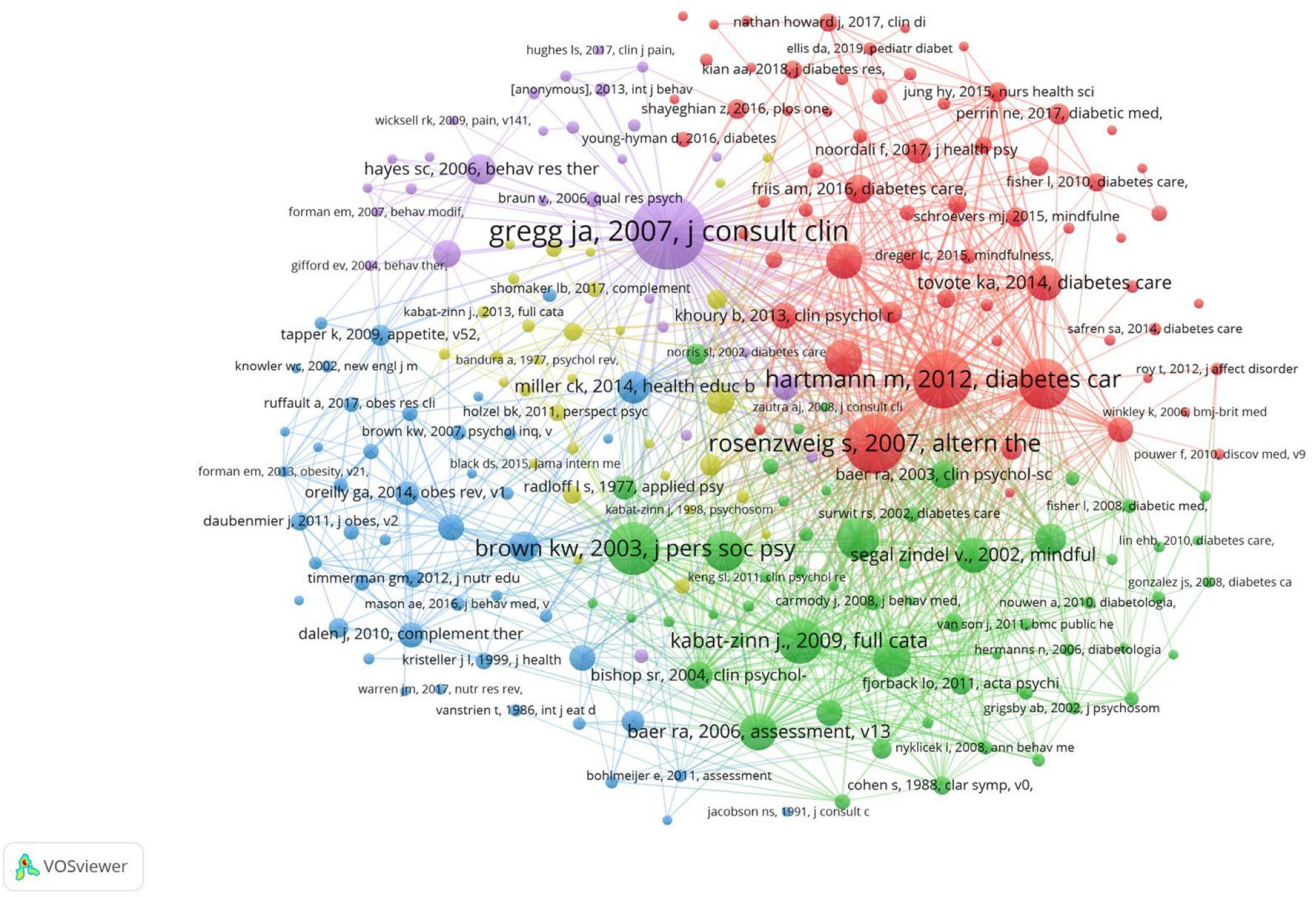
Reference co-citation analysis network diagram from 2005 to 2023

Analyzing co-citations in references is a crucial method for uncovering the structure and evolutionary path of a specific domain [36]. Figure 5 displays the top 228 references out of a total of 15,402, while Table 6 presents the top 10 most-cited references. Notably, Baer et al. made significant contributions with two meta-analyses [37, 38]. Furthermore, three pieces of literature [29, 39, 40] have affirmed the advantages of mindfulness therapy in improving the mental health of diabetic patients through randomized controlled trials.

**Table 6:** The top 10 cited references

Label	Title	Journal	Citations	Total link strength
Gregg (2007)	Improving diabetes self-management through acceptance, mindfulness, and values: a randomized controlled trial	Journal of Consulting and Clinical Psychology	61	749
Hartmann (2012)	Sustained effects of a mindfulness-based stress-reduction intervention in type 2 diabetic patients design and first results of a randomized controlled trial (the Heidelberger diabetes and stress-study)	Diabetes Care	45	637
brown (2003)	The benefits of being present: mindfulness and its role in psychological well-being	Journal of Personality and Social Psychology	40	428
Van Son (2013)	The effects of a mindfulness-based intervention on emotional distress, quality of life, and HbA(1c) in outpatients with diabetes (DiaMind)	Diabetes Care	38	560
Grossman (2004)	Mindfulness-based stress reduction and health benefits: a meta-analysis	Journal of Psychosomatic Research	30	330
Cohen (1983)	A global measure of perceived stress	Journal of Health and Social Behavior	29	269
Baer (2006)	Using self-report assessment methods to explore facets of mindfulness	Assessment	26	351
Hofmann (2010)	The effect of mindfulness-based therapy on anxiety and depression: a meta-analytic review	J Consult Clin Psychol	26	422
Kabat (2003)	Mindfulness-based interventions in context: past, present, and future	Clinical Psychology: Science and Practice	26	334
Van Son (2014)	Mindfulness-based cognitive therapy for people with diabetes and emotional problems: long-term follow-up findings from the DiaMind randomized controlled trial	Journal of Psychosomatic Research	25	401

In the 10 most cited articles, articles 2, 5, and 6 focus on research based on mindfulness-based stress reduction interventions. Articles 4, 8, 9, and 10 explore various strategies for diabetes mitigation (mindfulness interventions for emotional prediction) and their effects and potential mechanisms. The remaining articles emphasize mindfulness therapies for diabetes mitigation (self-management).

### Keyword analysis

Keywords play a vital role in encapsulating the essence of concentrated research in literature. The frequency with which keywords appear together can indicate the evolving trends in mindfulness intervention within the diabetes field. High-frequency keywords shed light on the research focus, advanced methodologies, pressing issues, or noteworthy academic subjects within a given time frame [41].

#### Keyword co-occurrence network analysis

**Fig. 6:**
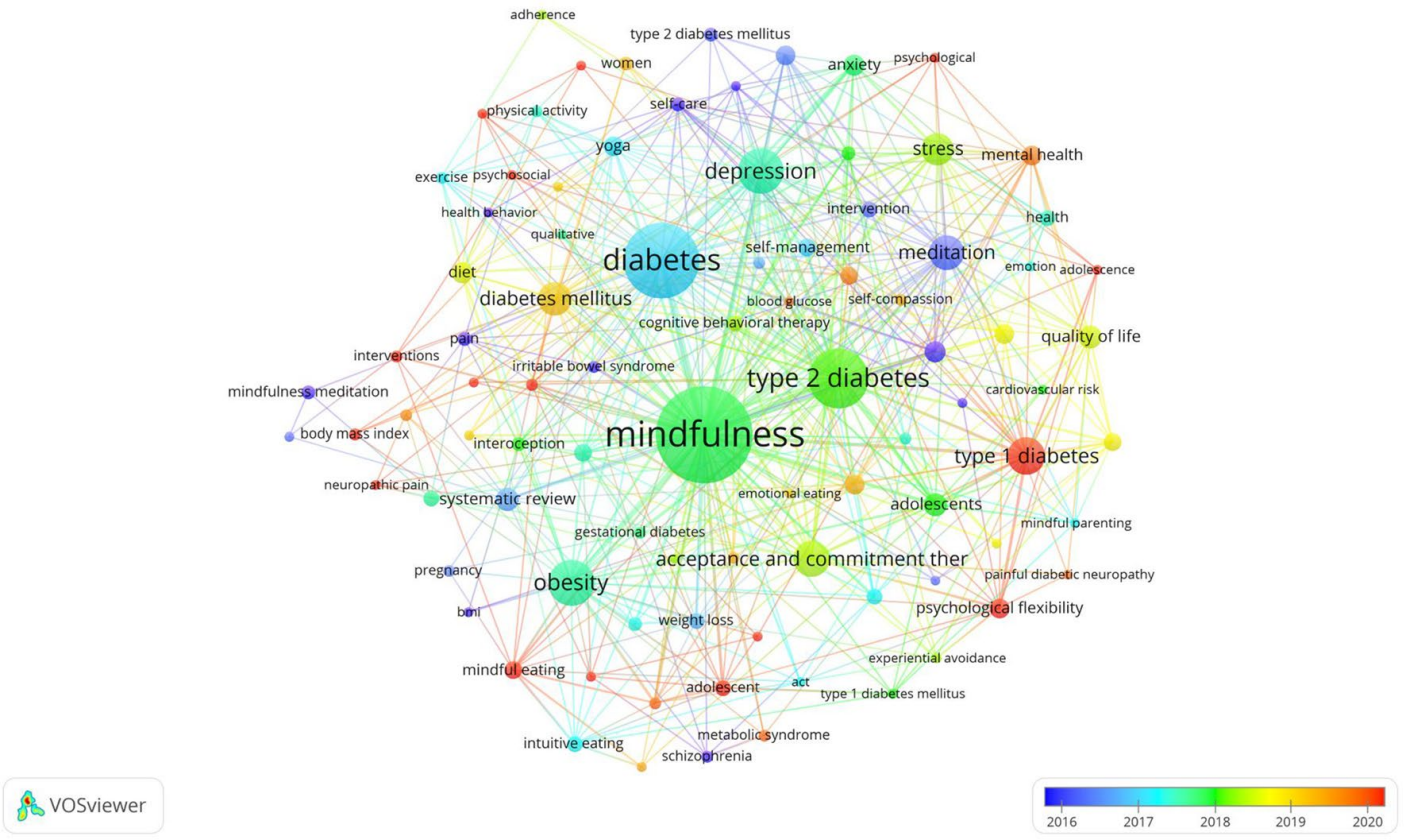
Network diagram of keyword co-occurrence analysis from 2005 to 2023

Figure 6 illustrates the network diagram for keyword co-occurrence analysis, encompassing all keywords considered in the analysis. We have displayed only the first 90 keywords out of 886 for analysis and discussion to maintain clarity and visual appeal. In Table 7, you will find the top 20 co-occurrence keywords, with “mindfulness” and “diabetes” being the most frequently encountered, followed by “type 2 diabetes,” “obesity,” “depression,” and “type 1 diabetes.” The recurring presence of keywords like “acceptance and commitment therapy,” “meditation,” “quality of life,” “anxiety,” and “mental health” signifies the widespread use of mindfulness-based therapies. “Mindfulness-based cognitive therapy” has been employed to enhance the self-management and quality of life of diabetes patients, with a specific emphasis on high-frequency keywords indicating that mindfulness-based therapy in diabetes primarily addresses mental health. Furthermore, a significant number of keywords associated with “randomized controlled trial studies” and “meta-analyses” have emerged in high frequency, signifying the reporting of empirical evidence regarding the clinical effectiveness of mindfulness interventions in diabetes.

**Table 7:** Top 20 keywords with frequency

Rank	Keywords	Occurrences	Total link strength
1	Mindfulness	77	200
2	Diabetes	54	104
3	Type 2 diabetes	39	84
4	Obesity	27	60
5	Depression	26	78
6	Type 1 diabetes	20	42
7	Acceptance and commitment therapy	19	42
8	Meditation	18	52
9	Diabetes mellitus	17	41
10	Stress	16	41
11	Adolescents	10	31
12	Quality of life	10	25
13	Systematic review	10	17
14	Anxiety	9	31
15	Diet	9	22
16	MBSR	9	25
17	Mental health	8	26
18	Meta-analysis	8	25
19	Psychological flexibility	8	20
20	Randomized controlled trial	8	22

#### Keyword clustering

**Fig. 7.**
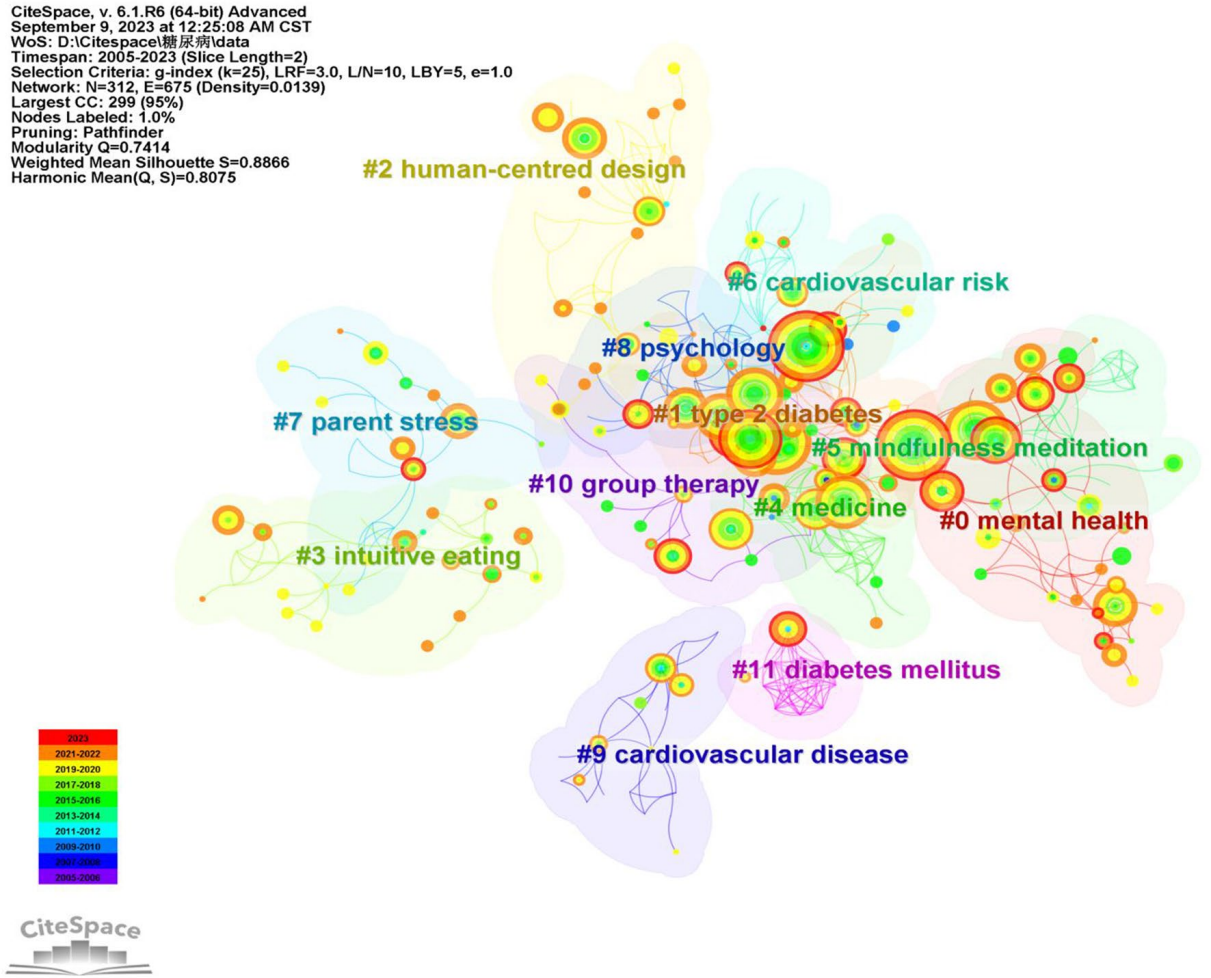
2005–2023 keyword clustering diagram

**Fig. 8.**
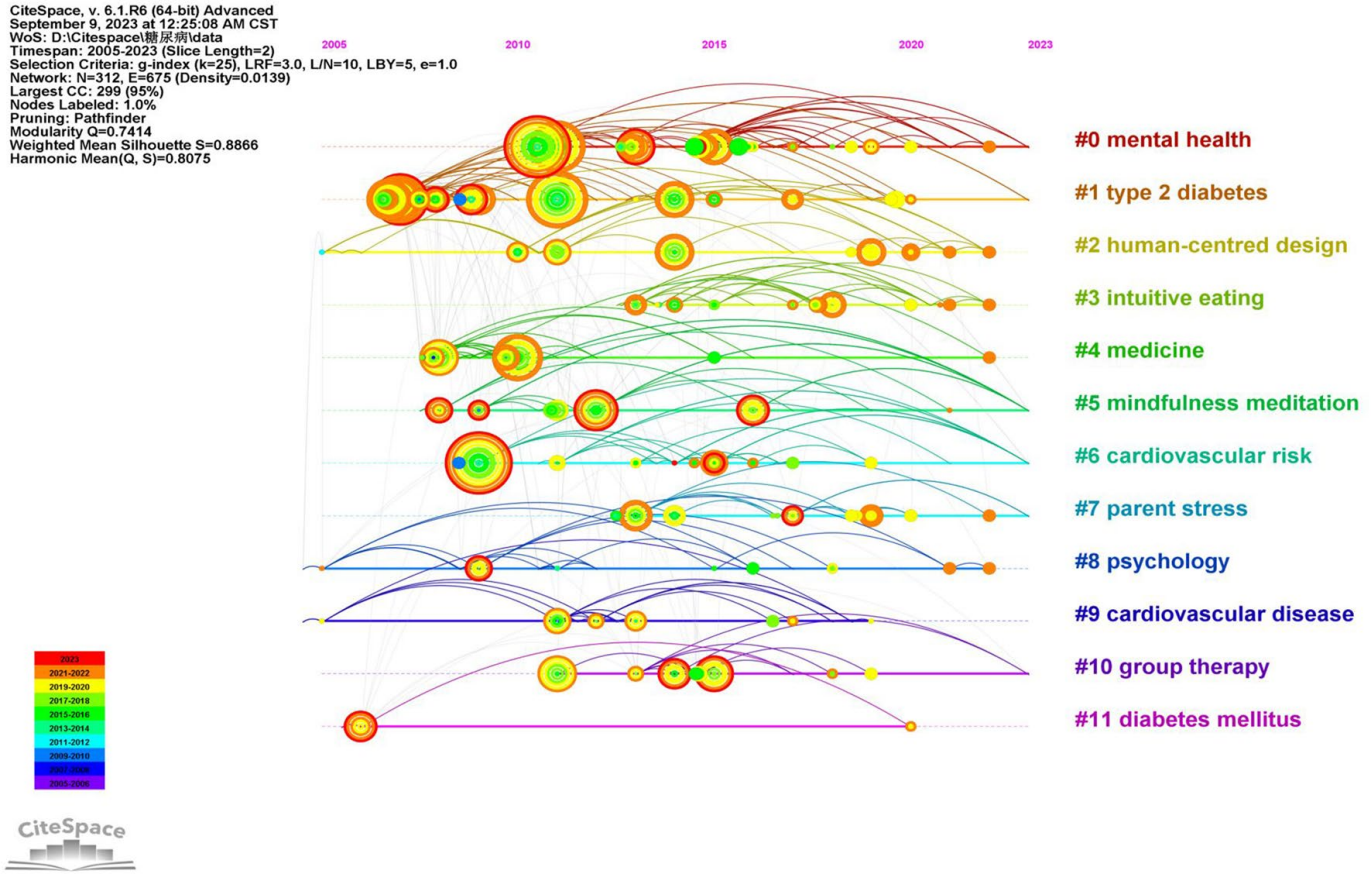
2005–2023 Keyword timeline map

Figure 7 illustrates the grouping of keywords based on their co-occurrence, alongside using the LLR log-likelihood algorithm to extract, label, and group keywords found in the cited literature. The results of keyword clustering in the study of mindfulness interventions for diabetes mellitus were highly significant (module value Q = 0.7414 > 0.3, contour value S = 0.8866 > 0.5), which holds substantial value for research and analysis. In Fig. 8, you will find the top 11 categories organized by the size of the clusters. This timeline network diagram, built upon keyword clustering, allows for a clearer display of the number of documents in each cluster. A larger number of documents in a cluster signifies the significance of that particular field and provides insights into each cluster’s temporal characteristics. The research hotspots are reflected in the timeline graph of keywords. It is evident that “mental health,” “human-centred design,” and “intuitive eating” have emerged as prominent focal points in recent years.

#### Keywords burst detection and analysis

The concept of keyword emergence delineates terms that become notably prominent and frequently occur within a specific timeframe, offering a means to predict shifts in research hotspots and future trends. Table 8 displays the top 30 keywords with the most significant burstiness from 2005 to 2023, among which “association” exhibits the strongest burst intensity. This indicates a broad academic focus on this term during 2018 to 2021. Given the necessity for interdisciplinary approaches in many contemporary research endeavors, “Association” is likely employed to denote interconnections across various domains. This trend towards interdisciplinary research may have catalyzed the increase in citation bursts for “Association”. The term “symptom” shows the longest duration of burstiness (9 years), highlighting it as a key research focus from 2007 to 2015. This may be indicative of researchers’ growing interest in the relationship between symptoms and health issues, encompassing symptom management and disease diagnosis across a spectrum of illnesses.

**Table 8:**
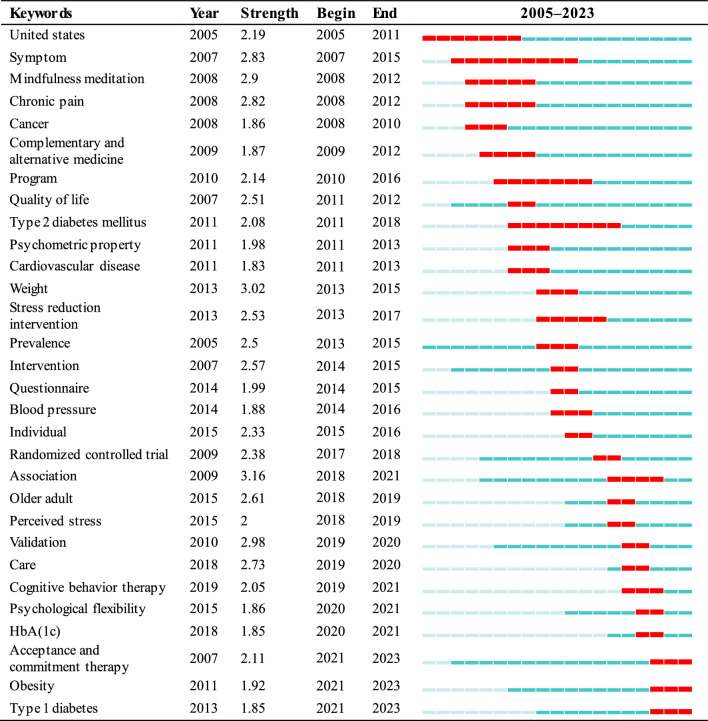
Top 30 keywords with the strongest citation bursts

Overall, the research trends demonstrate a diversified field encompassing health, mental health, geriatric health, chronic disease management, epidemiology, medical interventions, and treatment methods. These trends reflect the academic and societal continuous interest and investment in improving health and quality of life.

## Discussion

### Basic information

Between 2005 and 2010, the potential role of mindfulness interventions in diabetes management may not have been widely recognized. Over time, however, researchers and medical professionals began to take an interest in the potential benefits of mindfulness. As a result, the number of publications on mindfulness intervention in diabetes has increased between 2011 and 2023, becoming a new hot area. Diabetes is a global health problem, and mindfulness interventions are believed to improve patients’ quality of life and disease management. As more studies establish an association between mindfulness interventions and diabetes, researchers may be more willing to invest time and resources to delve deeper into this area. The accumulation of these research results has also attracted more scholars to participate in research. The medical community increasingly values research in this area and patients as it offers a non-pharmacological approach to intervention. Government, medical, and nonprofit support for diabetes management and mental health may prompt researchers to more actively explore the potential applications of mindfulness interventions. For these reasons, the literature on mindfulness interventions in diabetes is expected to continue to grow in the future.

Academic collaboration and knowledge sharing help to advance the field of research. Mindfulness intervention in diabetes research may benefit from international collaboration and interdisciplinary research that facilitates knowledge exchange. The United States is the most productive country for publications, accounting for 48% of all publications and 5872 citations, well ahead of the second-placed United Kingdom (862). Five of the top 10 institutions in the publication rankings are based in the United States, reflecting the country’s research activity in the field. The top three institutions are Tilburg University, Deakin University, and the University of North Carolina, which have made significant achievements in the study of mindfulness intervention in diabetes. The top three institutions for total citations are Tilburg University (564 citations), the University of California, San Francisco (551 citations), and Ohio State University (546 citations). In the collaborative network analysis, the United States, with a high degree of international activity, became the center of global cooperation between countries and regions in this field, followed by the United Kingdom and Australia. As a scientific research power, the United States has several high-yield research institutions in the field of diabetes management and mindfulness intervention research, which may be related to the advantages of the United States in scientific research resources, funding, and cooperation opportunities. Mindfulness intervention in diabetes research may be an international field, and collaboration and knowledge sharing may have played a positive role in institutional citations and outputs. The rankings and citations reflect the research dynamics within the field of mindfulness intervention in diabetes, as well as the excellence of some institutions in the field. The findings from these institutions have important implications for advancing the development of diabetes management and mindfulness interventions.

The journal mindfulness ranked first in the number of articles published in mindfulness intervention in diabetes, with 15 articles published on the subject. Other journals with a high number of publications include diabetes (12) and Diabetic Medicine (10). Diabetes Care ranked first in terms of total citations, reaching 416, making it the most cited journal. Mindfulness (317 citations) and Journal of Psychosomatic Research (312 citations) also performed well in ranking total citations. Diabetes Care ranked first in CA with a CA value of 104.00, indicating that the journal’s articles are relatively highly cited in the field. Among the top 20 journals, five articles have been cited more than 100 times. Mindfulness, Diabetes, and Diabetic Medicine are among the top journals in a number of published articles due to their focus on diabetes and mindfulness. These specialized journals may attract researchers to submit high-quality research. The journal focuses on high citations and CA values in a specific field. These journals may have played a key role in the development and promotion of the field of mindfulness intervention in diabetes. Some articles, such as Abbott et al.’s study, may be widely cited for their innovation and impact on mindfulness intervention in diabetes, driving research progress in the field.

### Global trends and research hotspots

“Symptom”, “quality of life”, “blood pressure”, “Mindfulness meditation”, “stress reduction intervention”, “intervention”, “randomized controlled trials”, “cognitive behavior therapy,” and “acceptance and commitment therapy” are keywords that burst over different periods. These keywords show that scholars are increasingly focusing on using mindfulness-related practices as proven ways to improve physical and mental health. They are also adopting more rigorous research designs to validate the effectiveness of these approaches.

#### The role of emotional intervention methods in the self-management of diabetes

In 2007, Gregg et al. [29] conducted a mindfulness-based randomized controlled trial with diabetes patients and found that patients who accepted mindfulness had better self-care skills. Tovote et al. [42] conducted a mindfulness-based randomized controlled trial on 94 diabetic patients with depression in 2014 and found that the depressive symptoms of the subjects were significantly reduced after the intervention. Schroevers et al. [43] found through a pilot study in 2015 that mindfulness-based cognitive therapy positively impacts the psychological improvement of diabetic patients. In a large cohort study of 1691 patients with T2DM, diabetes distress was positively associated with concurrent depression and persistent depression over time, while depression increased diabetes distress over time [44]. In addition, from 2013 to 2022, some scholars conducted systematic reviews and meta-analyses on the mental health, self-management, and physiological indicators of mindfulness intervention in diabetic patients [14, 30, 45, 46]. However, due to the low quality of some of the included studies, no clear and unified conclusions have been drawn. Therefore, we speculate that the effectiveness of mindfulness for diabetes will be further clarified based on high-quality RCTs in the future.

#### Research on metabolic diseases continues to receive significant attention

The keywords “hba (1c)”, “type 1 diabetes,” and “type 2 diabetes mellitus” indicate that research on metabolic diseases is still of great interest, especially about diabetes. As an important measurement index in diabetes research, hba (1c) has received scholars’ attention since 2013 [39]. Many RCT studies have also used “hba (1c)” as an outcome indicator to measure the effect of mindfulness interventions in patients with diabetes [47]. Secondary evidence [48, 49] shows mindfulness-based interventions effectively control blood glucose in adults with T1DM or T2DM. Overall, the current evidence on the effects of mindfulness on blood sugar control is often inconsistent, and the mechanisms of its effects may be discussed further in the future.

#### The impact of public health policy on obesity and cardiovascular diseases

Obesity has become one of the global public health issues [50]. The increasing prevalence of obesity worldwide will have a significant impact on the incidence of cardiovascular diseases, type 2 diabetes, and sleep apnea, among other illnesses [51]. Sedentary lifestyles, calorie-rich and low-nutrient foods, along with ongoing barriers to accessing care, are major contributors to obesity. Increasingly, obesity is recognized as an independent risk factor for the onset and mortality of cardiovascular diseases (CVD) [52]. In 2013, the NCCDPHP implemented a national public health action for the prevention of obesity, diabetes, heart disease, and stroke [53]. More and more evidence suggests that health approaches of various scales based on nutrition and physical activity may be a promising strategy for chronic disease prevention [54]. Therefore, the development and promotion of suitable public health policies can provide a theoretical basis for the field of chronic disease prevention and management and also offer reference value for national chronic disease programs.

#### Future, continued focus on the health of the elderly in chronic disease management

There have been keyword outbreaks in “older adult,” “chronic pain” and “psychological flexibility.” The risk of chronic diseases increases with age. Diabetes, as a kind of chronic disease with high incidence, accounts for a high proportion of the elderly. It can lead to a number of complications [55, 56]. Kayser [57] conducted a systematic review on the psychological outcomes of mindfulness intervention in elderly people with chronic diseases, and the results showed that mindfulness had certain positive effects on the mental health of elderly people with chronic diseases. Sayadi [58] conducted an intervention in the post-epidemic era of elderly people with diabetes in 2022, and the study showed that mindfulness training can effectively improve the anxiety, depression, and quality of life of elderly people with diabetes. With the global aging problem becoming more and more prominent, how to ensure the health and good mental state of the elderly has become a topic of concern. Future studies will need to explore the older population’s needs in greater depth to develop more targeted intervention and management plans. This could include developing more senior-friendly mindfulness training programs to maintain physical and mental health.

### Limitations

The software we used, including VOSviewer and CiteSpace, rely on publicly available literature databases (like the Web of Science we selected). Therefore, the research findings may be limited by the coverage and quality of these databases. Some fields of literature may not be included in these databases, leading to incomplete analysis. These tools typically use English-language literature databases so that they may overlook non-English literature. This could result in an incomplete understanding of global research trends. Errors, duplicates, or inaccuracies may exist in the databases, potentially misleading the analysis. Additionally, variations in author and institution names can introduce inaccuracies. Future research could further explore the domain of mindfulness interventions in elderly populations for the management of chronic diseases, particularly focusing on studies related to diabetes. This would aid researchers in gaining a better understanding of the field, its developments, and current trends.

## Conclusions

Our econometric analysis of the literature on mindfulness intervention in diabetes from 2005 to 2023 found an overall upward trend in annual publications, with the number increasing year by year. The United States is the country with the most publications and is the center of collaboration in this area of research. Tilburg University, Deakin University, and the University of North Carolina were the top three institutions in the publication rankings. The high number of literature outputs from these institutions in this field shows their influence in the research field. Cooperation between different countries and institutions should be strengthened to promote the application of mindfulness in the field of diabetes dry clinical pre-preparation. Currently, research in this area focuses on the effects of mindfulness on the mental health of people with diabetes. At the same time, the health management of elderly patients and the mechanism of metabolic diseases are also the focus of research in this field. These findings can provide a valuable reference for future research on mindfulness intervention in diabetes.

## Data Availability

Data can be obtained from the corresponding author upon reasonable request.
